# Entanglement detection with artificial neural networks

**DOI:** 10.1038/s41598-023-28745-3

**Published:** 2023-01-28

**Authors:** Naema Asif, Uman Khalid, Awais Khan, Trung Q. Duong, Hyundong Shin

**Affiliations:** 1 Department of Electronics and Information Convergence Engineering, Kyung Hee University, Yongin, Republic of Korea; 2 School of Electronics, Electrical Engineering and Computer Science, Queen’s University, Belfast, UK

**Keywords:** Quantum information, Qubits

## Abstract

Quantum entanglement is one of the essential resources involved in quantum information processing tasks. However, its detection for usage remains a challenge. The Bell-type inequality for relative entropy of coherence serves as an entanglement witness for pure entangled states. However, it does not perform reliably for mixed entangled states. This paper constructs a classifier by employing the relationship between coherence and entanglement for supervised machine learning methods. This method encodes multiple Bell-type inequalities for the relative entropy of coherence into an artificial neural network to detect the entangled and separable states in a quantum dataset.

## Introduction

There has been considerable advancement in the emerging quantum information technologies that offer many promising applications in communication and computation. Similarly, significant progress has also been achieved in the inter-disciplinary field of quantum information and machine learning^1^. There are two sides to this particular field of study. The first is using quantum information to improve classical machine learning algorithms. In contrast, the second corresponds to the use of classical machine learning algorithms to find innovative solutions to various challenges in quantum information science. Some of the methods that have already been proposed and implemented include techniques to solve the problem of quantum state preparation^2^, tomography^3^, quantum control^4^, and experiment searching^5^. Furthermore, research in quantum information for machine learning has also been investigated; some of which include entanglement for feature extraction^6^ and tensor network quantum states for supervised learning^7^.

Quantum entanglement, a peculiar property in quantum mechanics, enables us to achieve tasks impossible for classical systems. These tasks include ensuring secure communications and the speed-up of various hard computational tasks^8^. Therefore, an important question arises; “Given an unknown quantum state, how can we efficiently detect the presence of such a feature?” For high dimensional quantum systems, this is indeed a challenging task since quantum features usually indicate some correlated patterns concealed within sub-systems^9–11^. Typically, the most robust methods to detect entanglement involve full quantum state tomography. However, this method is experimentally demanding as the number of required projections increases with the number of qubits^12–14^. Entanglement can also be detected reliably through the positive partial transpose (PPT) criterion for lower-dimensional systems, that include the $2 \otimes 2$ and $2\otimes 3$ systems, but it is generically an NP-hard problem^15^.

Moreover, the aforementioned criteria fails to work for higher-dimensional quantum states, such as bound entangled states. In addition, other measures for the detection and quantification of entanglement have been presented, such as the covariance matrix criterion and the concurrence criterion. However, these also come along with their set of limitations^16–19^.

To find the solution to this problem, many researchers have turned to machine learning techniques capable of extracting features and recognizing patterns hidden in large-dimensional datasets. Several methods have been devised and tested for the problem of entanglement detection^20–24^. As performing full quantum state tomography becomes resource-consuming with the increase in the number of qubits, the concept of building classifiers using partial information of quantum states has been widely studied. For instance, training Bell inequalities as entanglement witnesses with artificial neural networks provide a suitable classifier^25^. The extended version of Bell’s inequality, i.e., Mermin’s inequality, also provides favorable results for the case of tripartite quantum states and bound entangled states^26,27^. Deep quantum neural network techniques have also been demonstrated to detect entanglement in high-dimensional quantum states^28^.

In addition, other machine learning techniques such as support vector machines and decision trees can also serve the purpose of building an entanglement-separability classifier^29^. Comparative studies between artificial neural networks and witness-based methods for classifying quantum states have demonstrated that artificial neural networks perform significantly better than witness-based methods^30^. Unsupervised learning techniques have also been studied for quantum state classification along with supervised learning. These techniques have also successfully detected entanglement in a multipartite quantum dataset^31^.

Another property of a quantum state, a basis dependant quantity, coherence, is identified by the presence of the off-diagonal terms in the density matrix representation of a quantum state. Many coherence measures have been proposed to quantify the coherence in a given state. These include the $l_1$ norm of coherence and relative entropy of coherence^32^. The relationship between the two properties, entanglement, and coherence, has also been discussed^33^. Inequality based on Bell’s inequality and the relative entropy of coherence has also been proposed^34,35^. These studies show that coherence and entanglement have some intricate relationships between them for different classes of quantum states.

**Figure 1:**
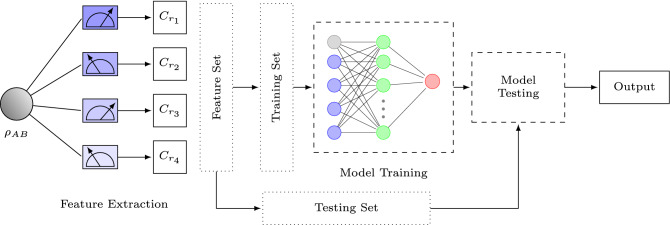
Coherence-based entanglement detection with artificial neural networks.

This work investigates the classification of quantum states by designing a classifier using an artificial neural network. We extract features from the quantum states using the terms of the Bell-type inequality for relative entropy of coherence. The Bell-type inequality of the relative entropy of coherence shows the relation between coherence and entanglement of quantum states, so the classifier encodes both properties for reliable detection of entanglement and classification of quantum states. Moreover, as we use partial information of a quantum state as a feature set, quantum state tomography is not required. Since a single entanglement witness cannot operate on several states, we encode multiple Bell-type inequalities for relative entropy of coherence into the artificial neural network classifier for the reliable classification of quantum states. Our idea is to provide our classifier with a large amount of sample data with their corresponding labels and then test the classifier to predict the label of new states that it has not encountered before. Furthermore, we demonstrate the significant increase in accuracy and capability of a neural network-based entanglement-separability classifier by invoking a hidden layer. The approach discussed in the paper for quantum entanglement detection is shown in the Fig. 1.

The paper is organized as follows. We explain the model and methods used in the paper, such as the Bell-type inequality for relative entropy of coherence, data generation, model training, and testing. Furthermore, we explain the results obtained by the experiment. Finally, we conclude the paper.

## Methods

This section introduces a method of coherence-based entanglement detection with an artificial neural network. Typically, entanglement detection methods require partial information about the underlying quantum states. Herein, we obtain partial information about quantum states using relative entropy of coherence and Bell’s inequality. Then, we use this information to construct a classifier that reliably predicts unknown quantum states as either entangled or separable. Thus, the classifier employs the coherence properties of unknown quantum states to predict its entanglement properties.

### Optimizing Bell-type inequality for relative entropy of coherence with machine learning

An arbitrary quantum state $\rho$ consisting of *n* qubits is fully separable if we can express it as a convex combination of product states as

1 $$\begin{aligned} \rho _{\mathrm{sep}} = \sum _i p_i \, \rho ^1_i \otimes \rho ^2_i \otimes \ldots \otimes \rho ^n_i, \end{aligned}$$

where $0 \le p_i \le 1$ and $\sum _i p_i = 1$, otherwise the quantum state is entangled. Now, we consider the following basis

$$ Q : \{ \left| 0\right\rangle , \left| 1\right\rangle \} ,\; R: \{ \left| R_+\right\rangle , \left| R_-\right\rangle \}, \; S : \{ \left| S_+\right\rangle , \left| S_-\right\rangle \} ,\; T: \{ \left| 0\right\rangle , \left| 1\right\rangle \},$$

where,

$$\begin{aligned} \left| R_+\right\rangle&= \frac{1}{\sqrt{2}}(\left| 0\right\rangle + \dot{\iota } e^{\dot{\iota }\phi }\left| 1\right\rangle ),\\ \left| R_-\right\rangle&= \frac{1}{\sqrt{2}}(\left| 0\right\rangle - \dot{\iota } e^{\dot{\iota }\phi }\left| 1\right\rangle ),\\ \left| S_+\right\rangle&= \frac{1}{\sqrt{2}}(\left| 0\right\rangle + \left| 1\right\rangle ), \\ \left| S_-\right\rangle&= \frac{1}{\sqrt{2}}(\left| 0\right\rangle - \left| 1\right\rangle ). \end{aligned}$$

For these observables, we calculate the relative entropy of coherence given as $C_r(QS,\rho _{\mathrm{AB}}),\; C_r(RS,\rho _{\mathrm{AB}}),\;C_r(RT,\rho _{\mathrm{AB}}),$ and $C_r(QT,\rho _{\mathrm{AB}})$. Since coherence is a basis dependent quantity, we specify the basis we have chosen. In other words, we denote $C_r(QS,\rho _{\mathrm{AB}})$ by $C_r(\rho _{\mathrm{AB}})$ in the reference basis formed by *Q* and *S*. Herein, the measure of coherence based on relative entropy for a quantum state $\rho$ with reference basis $\{\left| i\right\rangle \}$ is defined as^32^

2 $$\begin{aligned} C_r(\rho ) = \min _{\sigma \in \Gamma } S(\rho || \sigma ) = S(\rho ^d) - S(\rho ), \end{aligned}$$

where we have $\Gamma$ as the set of all incoherent states in the reference basis $\{\left| i\right\rangle \}$. The relative entropy between $\rho$ and $\sigma$ is described as $S(\rho || \sigma ) = \mathrm{tr} \rho (\log { \rho }- \log {\sigma })$ while the von-Neumann entropy of $\rho$ is $S(\rho ) = - \mathrm{tr} \rho \log { \rho }$ . The diagonal state of $\rho$ is $\rho ^d$, that can be expressed as $\rho ^d = \sum _i{\left\langle i\right| \rho \left| i\right\rangle \left| i\right\rangle \left\langle i\right| }$.

The Bell-type inequality is formulated for relative entropy of coherence as^34^

3 $$\begin{aligned} C_r(QS,\rho _{\mathrm{AB}}) + C_r(RS,\rho _{\mathrm{AB}})+ C_r(RT,\rho _{\mathrm{AB}}) - C_r(QT,\rho _{\mathrm{AB}}) \le 4. \end{aligned}$$

This inequality is generally satisfied for separable states and violated for entangled states, for the considered set of observables. However, many cases exist where quantum states do not violate the inequality even though it is entangled^34^. Therefore, for the above inequality to perform as a classifier, we introduce weight factors on each of the terms and obtain the following weighted equation for the relative entropy of coherence

4 $$\begin{aligned} {\begin{matrix} \Pi _{\mathrm{ML}} = w_0 + w_1 C_r(QS,\rho _{\mathrm{AB}}) + w_2 C_r(RS,\rho _{\mathrm{AB}}) + w_3 C_r(RT,\rho _{\mathrm{ AB}}) + w_4 C_r(QT,\rho _{\mathrm{AB}}) , \end{matrix}} \end{aligned}$$

where the weights $w_0, w_1, w_2, w_3,$ and $w_4$ are obtained by training the machine learning models, namely, artificial neural networks. For a given quantum state, the set of the following outcomes

$$\{ C_r(QS,\rho _{\mathrm{AB}}),\; C_r(RS,\rho _{\mathrm{AB}}),\;C_r(RT,\rho _{\mathrm{AB}}), \;C_r(QT,\rho _{\mathrm{AB}})\;\},$$

is taken as the features of the supervised machine learning model. Since we focus on bipartite systems, the labels are obtained via the PPT criterion^15^. We observe that the performance relies heavily on the testing data, and the primary source of error arises from the data near the boundary between the separable and the entangled states.

### Generating labeled quantum datasets

This section describes different procedures used to generate quantum data. For the first procedure, we generate 50, 000 quantum states by using random values of $\theta$ and $\phi$ in the following

5 $$\begin{aligned} \rho _{\theta ,\phi } = p\left| \psi _{\theta ,\phi }\right\rangle \left\langle \psi _{\theta ,\phi }\right| + (1-p)\frac{I}{4}, \end{aligned}$$

where $\left| \psi _{\theta ,\phi }\right\rangle$ is given as follows

6 $$\begin{aligned} \left| \psi _{\theta ,\phi }\right\rangle = \cos \left( \frac{\theta }{2}\right) \left| 01\right\rangle + e^{\iota \phi }\sin \left( \frac{\theta }{2}\right) \left| 10\right\rangle . \end{aligned}$$

We also generate 50, 000 quantum states of the following family and use (5) to introduce noise

7 $$\begin{aligned} \left| \psi _{\theta ,\phi }\right\rangle = \cos \left( \frac{\theta }{2}\right) \left| 00\right\rangle + e^{\iota \phi }\sin \left( \frac{\theta }{2}\right) \left| 11\right\rangle . \end{aligned}$$

For the second procedure, 50, 000 entangled states are generated by random density matrices, whereas 50, 000 separable states are generated by taking the product of two separately generated random density matrices. In another procedure, 50, 000 pure quantum states are generated and are mixed by an arbitrary noise factor according to (5), where $\left| \psi _{\theta ,\phi }\right\rangle$ denotes the pure state. In addition to the above procedures, we generate 50, 000 samples of each of Bell states $\left| \psi _{+}\right\rangle$ and $\left| \phi _{+}\right\rangle$ affected by a random noise factor of *p* as in (5) where $\left| \psi _{\theta ,\phi }\right\rangle$ refer to the Bell states in (8) and (9), respectively.

8 $$\begin{aligned} \left| \psi _{+}\right\rangle= & {} \frac{1}{\sqrt{2}}\left( \left| 01\right\rangle + \left| 10\right\rangle \right) , \end{aligned}$$

9 $$\begin{aligned} \left| \phi _{+}\right\rangle= & {} \frac{1}{\sqrt{2}}\left( \left| 00\right\rangle + \left| 11\right\rangle \right) . \end{aligned}$$

In this way, we consider many quantum state families while building the classifier, therefore working towards a more general solution. The total size of the generated data consists of 350, 000 samples. The quantum states in all the methods mentioned above are generated using the functions of the QETLAB package used in MATLAB to explore the theory of quantum entanglement^36,37^.

We can detect the state as entangled or separable for a bipartite state using the PPT criterion^15^. Therefore, we define $\rho ^{T_B}_{\theta ,\phi }$ as the matrix obtained by taking the partial transpose of $\rho _{\theta ,\phi }$ in the second qubit. The label of a quantum state is taken as 1, i.e., entangled, if the smallest eigenvalue of the density matrix $\rho ^{T_B}_{\theta ,\phi }$ comes out to be negative. Otherwise, it is taken as 0, i.e., separable.

For the generated dataset, the labels are obtained by using the PPT criterion function available in the QETLAB package, which automatically calculates the partial transpose and computes the minimum eigenvalue to determine whether the quantum state in question is entangled or separable^36,37^. Furthermore, we extract the required features given by the relative entropy of coherence, as discussed before. We use this dataset to build the most suitable classifier by training our machine learning model.

**Figure 2:**
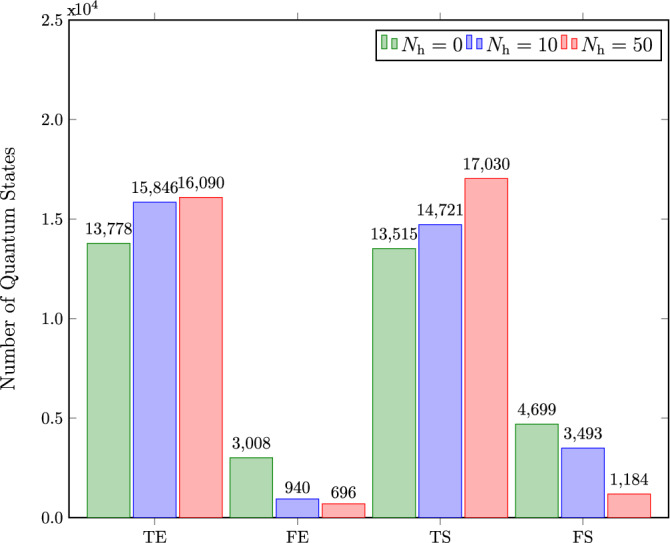
Bipartite quantum states classified as truly entangled (TE), falsely entangled (FE), truly separable (TS), and falsely separable (FS) by testing classifiers with $N_\mathrm{h} = 0$, $N_\mathrm{h} = 10$, and $N_\mathrm{h} = 50$, where $N_\mathrm{h}$ denotes the number of neurons in the hidden layer.

### Training the artificial neural network

We utilize the artificial neural network to design a classifier as our machine learning method. The generated dataset is loaded and divided into $70\%$ training and $30\%$ test sets. We construct and train the simplest neural network consisting of linear connection and non-linear output with sigmoid as its activation function. We use the loss function given by the binary cross-entropy and the RMSprop optimizer with default hyperparameters. In addition, accuracy metrics are used to observe the neural network’s performance. Callbacks are used by monitoring the value of validation loss to obtain the best model. The model is trained for 100 epochs and tested by the datasets to get the linear weights.

Furthermore, we improve the network’s accuracy by inserting a hidden layer to introduce non-linearity. ReLu function is taken as activation function for the hidden layer nodes while sigmoid function for the output layer node. Moreover, multiple models are trained for the number of hidden neurons in the set $\{0,5,...,50\}$ for the same dataset. This step highlights the accuracy trend with the increase in neurons. Here, all the weights are initialized uniformly and are optimized through the learning process. We implement these neural networks using Keras and TensorFlow in a Jupyter notebook environment^38^.

## Numerical results

In this section, we discuss our findings and numerically analyze our proposal. To demonstrate the machine learning improvement, we first use the inequality as an entanglement witness on our dataset. By this test, we obtain an accuracy of $49.12\%$, with 178, 048 samples out of the total 350, 000 being predicted as falsely separable. These results show that the entanglement witness has a significant value of type-II error. After training the neural networks with the generated dataset, we test with the testing set and newly generated data to obtain the performances of the machine-learned classifiers.

### Testing with general testset

On testing the classifiers, we observe that the linear optimization by the simple neural network gives us an accuracy of $78.18\%$ for our data. By introducing a hidden layer, i.e., non-linear optimization, we observe a drastic increase in accuracy to $94.62\%$ with 50 hidden neurons. Having hidden neurons more than 50 shows no further increase in accuracy value. To further elaborate our results, we obtain the confusion matrices by testing the classifiers. Figure 2 depicts these results. By introducing non-linearity in our model, the falsely entangled samples decrease from 4699 to 1184 samples, whereas the falsely separable samples change from 3008 to 696 samples. Hence, this proves that the classifier constructed by the hidden layer neural network outperforms the neural network with no hidden layer.

Moreover, we observe the performance of the classifier by training the models on different quantum dataset sample size. On increasing the dataset size from $10^3$ to $10^5$, the test accuracies of the model increase as given in Table 1.

**Table 1:** Effect on accuracy with the increase of sample size.

Sample Size	$N_{\mathrm{h}} = 0$	$N_{\mathrm{h}} = 5$	$N_{\mathrm{h}} = 10$	$N_{\mathrm{h}} = 50$
$10^3$	74.0	74.5	75.0	80.0
$10^4$	75.4	82.3	85.85	87.50
$10^5$	77.86	84.56	88.33	94.07

**Figure 3:**
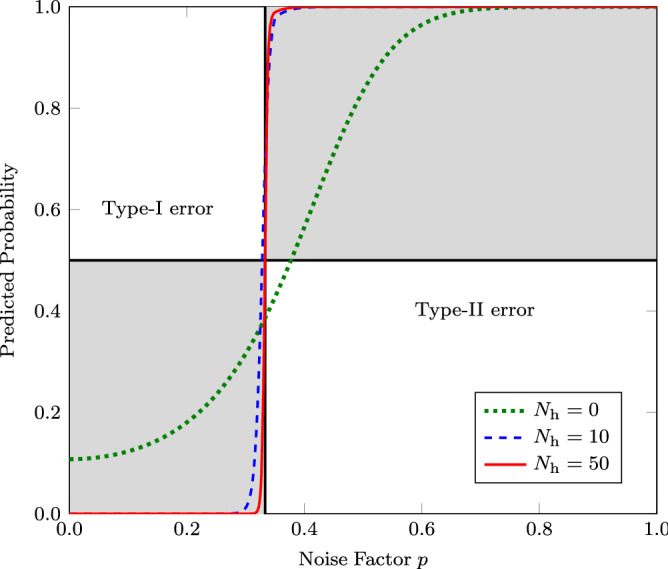
Probability of a quantum state in Eq. (10) being predicted as either entangled or separable as a function of the noise factor *p*. Here $N_\mathrm{h}$ denotes the number of neurons in the hidden layer. Truly entangled and truly separable regions are represented by shaded regions. This also highlights the effect on detection accuracy with the increase in the number of hidden-layer neurons.

### Comparison with entanglement witness

We test the trained classifier using a newly generated dataset based on the quantum states given by

10 $$\begin{aligned} \rho = p\left| \psi \right\rangle \left\langle \psi \right| + (1-p)\frac{I}{4}, \end{aligned}$$

where $p \in (0,1)$ and is known as the noise factor and $\left| \psi \right\rangle$ is a bell state given as

11 $$\begin{aligned} \left| \psi \right\rangle = \frac{1}{\sqrt{2}}(\left| 00\right\rangle + \left| 11\right\rangle ). \end{aligned}$$

We observe how well the classifier performs depending on the noise level corresponding to the value of *p*. The states mentioned are separable states for $p < 1/3$ and are entangled if the value exceeds 1/3. We observe that a model without hidden neurons, i.e., linear optimization, can yield a significant amount of samples classified as falsely separable, as shown in Fig. 3. So the model exhibits type-II error. The learning model with 10 neurons shows partial improvement. However, some samples are classified as falsely entangled, as indicated by the blue dashed line in the Fig. 3, causing the model to display a type-I error. In our model, we can observe a significant gain in the classifier’s performance as we further increase the number of neurons to 50.

In the proposed method, we observe that employing the original inequality as an entanglement witness results in a high value of type-II error with an accuracy of $49.12\%$. However, the classifier trained with the simplest neural network classifies quantum states with an accuracy of $78.18\%$, significantly reducing type-II errors. Also, introducing a hidden layer improves the classification performance and predicts the results with an accuracy of $94.62\%$. This shows a significant improvement compared to the original inequality as an entanglement witness. It is because the classifier built with the artificial neural network encodes multiple entanglement witnesses, which work simultaneously to detect entanglement better than a single entanglement witness.

### Detecting entanglement in tripartite quantum states

**Figure 4:**
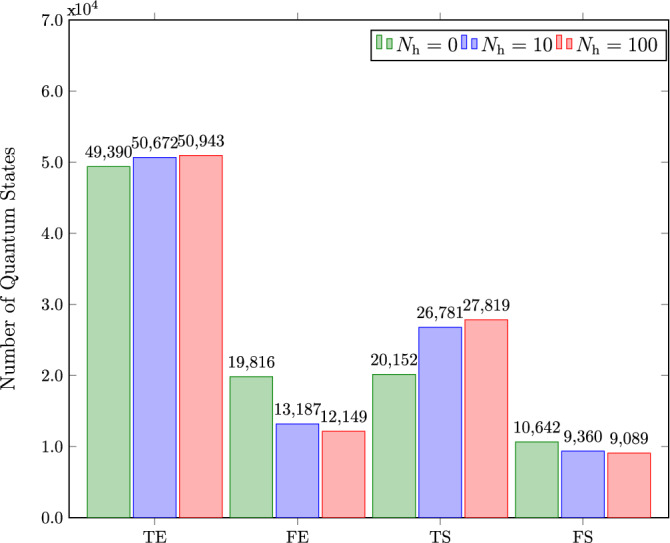
Tripartite quantum states classified as truly entangled (TE), falsely entangled (FE), truly separable (TS), and falsely separable (FS) by testing classifiers with $N_\mathrm{h} = 0$, $N_\mathrm{h} = 10$, and $N_\mathrm{h} = 100$, where $N_\mathrm{h}$ denotes the number of neurons in the hidden layer.

In order to scale up our method to a tripartite quantum system, we generate a three-qubit dataset and extract the features according to the Bell-type inequality for tripartite states given in^35^

12 $$\begin{aligned} C_r(a^\prime bc,\rho _{\mathrm{ABC}}) + C_r(ab^\prime c,\rho _{\mathrm{ABC}})+ C_r(abc^\prime ,\rho _{\mathrm{ABC}}) - C_r(a^\prime b^\prime c^\prime ,\rho _{\mathrm{ABC}}) \le 6, \end{aligned}$$

where $\rho _{\mathrm{ABC}}$ denotes the density matrix of a tripartite quantum state and $\lbrace (a,a^\prime ),(b,b^\prime ),(c,c^\prime )\rbrace$ are the basis for the three qubits respectively. In this case, we consider fully separable states as one class and biseparable and fully entangled states as the other class for binary classification. After training several ANN models, we observe that the accuracy for the ANN having a single hidden layer with 100 neurons gives us an accuracy of $78.76\%$. By employing a model with three hidden layers, this accuracy improves upto $80.2\%$. We obtain the confusion matrices in Fig. 4 by testing the classifiers with hidden neurons in the set $\lbrace 0,10,100\rbrace$. The results show that the method also applies to tripartite quantum states.

However, compared to the bipartite state classification, we observe that four features are insufficient to achieve high accuracy for tripartite state classification. It is depicted by the relatively small increase in the correctly labeled samples in Fig. 4. Therefore, to increase the classifier’s performance, we have to increase the number of features for model training and modify the neural network architecture. In other words, the trade-off between achievable accuracy and resources becomes evident for a quantum system’s increased number of qubits. On the other hand, this method is computationally less expensive than quantum state tomography as we use partial information in which the number of features required is less than the number of terms in a quantum state density matrix.

## Conclusions

In this work, we have designed a classifier for detecting quantum states as separable and entangled using supervised learning and Bell-type inequality for relative entropy of coherence. We have generated a quantum dataset and have observed that using the Bell-type inequality as an entanglement witness on our quantum dataset gives us an accuracy of $49.12\%$ having a large amount of type-II error. In order to obtain better performing classifiers, we have chosen an artificial neural network as our machine learning method and trained it on a quantum dataset generated by linear optimization, i.e., a simple neural network (no hidden layer), and non-linear optimization, i.e., a neural network with at least one hidden layer. The classifier trained with the simplest neural network distinguishes the quantum states with an accuracy of $78.18\%$. Furthermore, we have observed a significant increase in the classifier’s performance on increasing the number of neurons and have observed that for our data, an artificial neural network with 10 to 50 hidden neurons serves the purpose and predicts the results with an accuracy of $94.62\%$. In this way, we have obtained a classifier that detects the entanglement using the coherence of a quantum state. We have used this classifier to observe its performance on Bell states affected by the noisy channel and have obtained favourable results. Moreover, we have tested our method for classifying tripartite quantum states and have observed that this approach can be extended to multipartite systems as well. However, the required number of features has to be increased along with the modification of the neural network. Our results pave the way for devising reliable entanglement detection tools for applications in quantum communication and computation.
